# CDK4/6 Inhibitors as Upfront Treatment in a Patient with Breast Cancer Presenting with a Clinical Critic Situation: A Case Report and Review of the Literature

**DOI:** 10.3390/curroncol29120756

**Published:** 2022-12-06

**Authors:** Giada Targato, Lucia Bortot, Arianna Dri, Marta Bonotto, Alessandro Marco Minisini, Gianpiero Fasola, Mauro Mansutti

**Affiliations:** 1 Department of Medicine (DAME), University of Udine, 33100 Udine, Italy; 2 Department of Oncology, Udine Academic Hospital, Azienda Sanitaria Universitaria Friuli Centrale (ASUFC), 33100 Udine, Italy

**Keywords:** breast cancer, chemotherapy, endocrine therapy, CDK4/6 inhibitors, Abemaciclib, visceral crisis

## Abstract

CDK4/6 inhibitors have revolutionized the treatment algorithm of luminal metastatic breast cancer, becoming the recommended first-line therapy in association with endocrine therapy. However, due to its theoretically greater and more rapid tumor shrinkage, the upfront use of chemotherapy is considered in some clinical situations like visceral crisis. At the state of the art level, a paucity of data is available about the use of CDK4/6 inhibitors in patients presenting with visceral crisis or with life-threatening conditions since this population was historically excluded from clinical trials. In addition, data regarding direct comparison between combinations of chemotherapy and CDK4/6 inhibitors in terms of efficacy, rapidity of responses and long-term outcomes are lacking. We report the case of a 68-year-old woman with luminal metastatic breast cancer presenting at diagnosis with a critical and potentially life-threatening condition. The patient was treated with first-line Abemaciclib plus letrozole and achieved a rapid partial response with sudden clinical stabilization. Although the patient did not technically present with a visceral crisis, this case presentation also endorsed the upfront use of CDK4/6 inhibitor combinations in critical clinical situations in the absence of severe organ dysfunction and after multidisciplinary discussion.

## 1. Introduction

Approximately 5–8% of breast cancer (BC) patients present with distant metastases at the time of diagnosis [1]. De novo metastatic BC is a highly heterogeneous disease that encompasses a range of different clinical situations and prognosis, according to burden of disease, sites of metastases, histotype and molecular subtype. Stage IV BC remains an incurable condition; however, dramatic therapeutic improvements have been achieved in the last decade.

Regarding hormone receptor (HR)-positive, human epidermal growth factor receptor 2 (HER2)-negative advanced BC, several studies highlighted significantly improved overall responses and long-term outcomes with the combination of CDK 4/6 inhibitors plus endocrine therapy (ET) compared to ET alone, irrespective of menopausal status [2,3,4,5,6,7,8,9,10,11,12]. Therefore, this strategy has become the new therapeutic standard, endorsed by the main international guidelines as first-line treatment for ER-positive and HER2-negative metastatic BC, in both endocrine sensitive and resistant settings [13,14]. There are no large, prospective, randomized studies which provide a head-to-head comparison between chemotherapy (CT) and CDK 4/6 inhibitor-based therapy as first-line treatment in de novo metastatic disease. However, the clinical use of CT as upfront strategy today is limited to situations including the presence of visceral crisis, which occurs in around 10–15% of de novo metastatic BC cases. The concept of visceral crisis was oftentimes revisited in recent years and nowadays there is not a widely accepted definition: according to the ESO-ESMO international consensus guidelines (ABC 5), it could be defined as a severe organ dysfunction, which involves severe symptoms, laboratory values alteration and rapid disease progression due to the presence of visceral metastases associated with a life-threatening organ compromise [14]. Therefore, it requires a rapid and reliable effective therapy with a manageable safety profile and a tailored dosing schedule. CT is supposed to be associated with greater and earlier tumor shrinkage and symptom control, especially in cases of high burden of visceral disease [15,16]. Limited data are available about the activity of CDK4/6 inhibitors in patients with visceral crises since this population was excluded from clinical trials. We presented the clinical case of a post-menopausal woman diagnosed with de novo luminal-like metastatic BC, highly symptomatic and with a life-threatening condition, who received ET in association with a CDK4/6 inhibitor as first-line treatment.

## 2. Case Presentation

A 68-year-old woman accessed the Emergency Department of Udine Academic Hospital on the 31st of March 2021 for severe dyspnea, cough and asthenia with sudden worsening over the past week. The patient had no other relevant comorbidities, except for pharmacologically controlled hypertension, and she never smoked.

**Figure 1:**
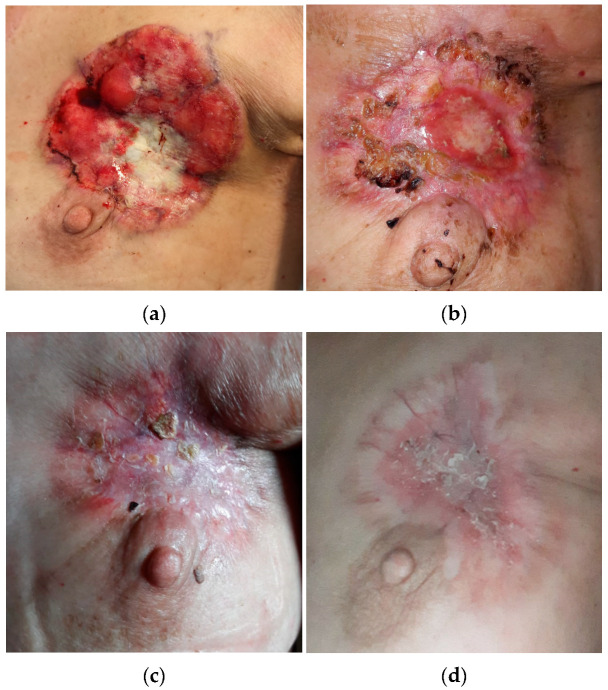

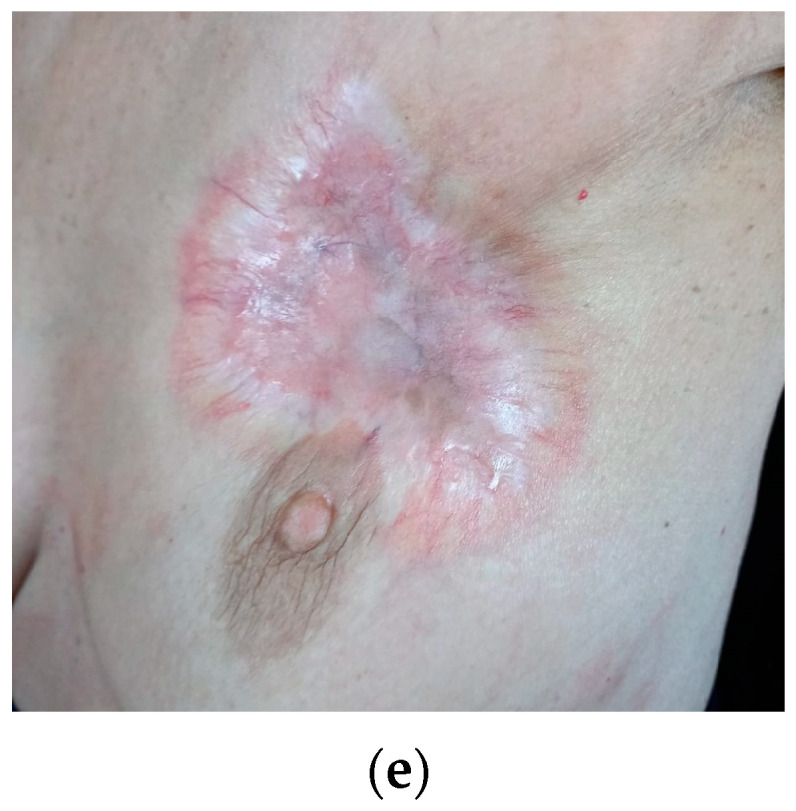
Baseline clinical evaluation and response over time of the primitive ulcerative breast lesion: (**a**) Clinical picture March 2021: left breast primitive neoplasm presenting as an ulcerative and bleeding lesion occupying the upper quadrants, about 10 × 70 mm in size; (**b**) Clinical picture May 2021: initial size reduction of the primary left breast neoplasm, in particular with respect to the actively bleeding area; (**c**) Clinical picture August 2021: reconstitution of intact skin, disappearance of bleeding and ulcerated areas; (**d**) Clinical picture November 2021: further dimensional reduction of the primitive left breast lesion, complete recovery of skin integrity; (**e**) Clinical picture March 2022 at one year from diagnosis.

At medical evaluation, the patient presented with pale skin and low blood pressure with high heart rate, while a detailed physical examination highlighted an ulcerative and bleeding lesion entirely occupying the upper quadrants of the left breast, with an estimated overall size of 10 × 7 cm (Figure 1a). Pathological lymphadenopathies were perceivable in ipsilateral axillary cavity and supraclavicular areas, having a major diameter of 5.5 cm and 4.0 cm, respectively. Blood tests revealed a hemoglobin value of 2.6 g/dL, likely due to chronic bleeding from the ulcerative lesion lasting for at least twelve months.

The patient was promptly admitted to the Internal Medicine Department for supportive therapy and received multiple blood transfusions, low-flow oxygen therapy for dyspnea and appropriate management of the ulcerated lesion.

**Figure 2:**
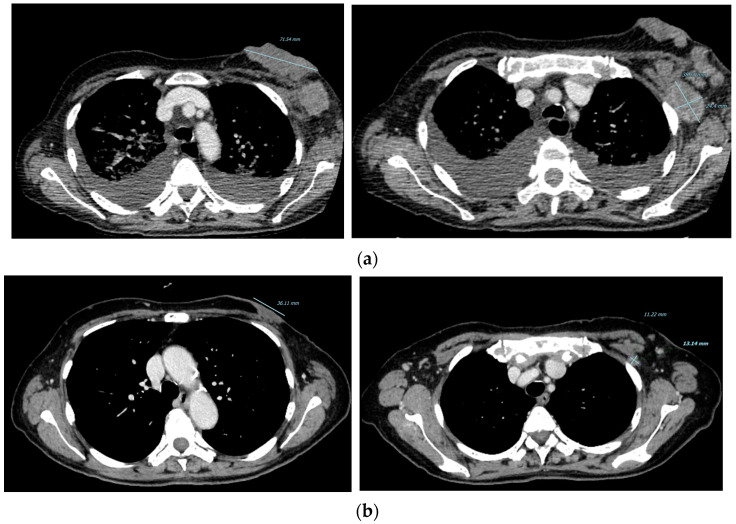
Baseline CT scan evaluation and subsequent time point: (**a**) CT scan March 2021, basal evaluation: left breast primitive lesion and left axilla lymphadenopathy; (**b**) CT scan November 2021: size reduction of left breast primitive lesion and left axilla lymphadenopathy.

Bilateral breast ultrasonography was performed with the results being pathognomonic of neoplasia, category 6, according to BIRADS classification [17]. Afterwards, the patient underwent excisional biopsy of the more-easily accessible left axillary lymph node, and subsequent histological and immunohistochemical examination confirmed diagnosis of ER-positive and HER2-negative non-special type BC (ER 100%, progesterone receptor (PgR) 80%, Ki67 30% and an intermediate degree of differentiation G2). Standard staging was completed with total-body computed tomography (TC) and bone scan, which revealed neoplastic involvement of almost the whole skeleton and absence of visceral metastasis (Figure 2a). None of the affected bone sites was considered at immediate risk of fracture and marrow failure was excluded since white blood cells and platelet levels were within the normal range.

After collegial evaluation and after reaching hemoglobin security levels with blood transfusions, on 10th of April 2021 standard first-line treatment for post-menopausal women with luminal BC was started, with the combination of letrozole 2.5 mg and Abemaciclib 300 mg per day continuously. This CDK4/6 plus ET combination was chosen since the every-day schedule is associated with better patient compliance and a lower risk of dosage mistakes. In addition, the patient underwent necessary dental care, in order to receive bisphosphonate treatment with three-monthly zoledronic acid, taking into account the high burden of bone disease. 

**Figure 3:**
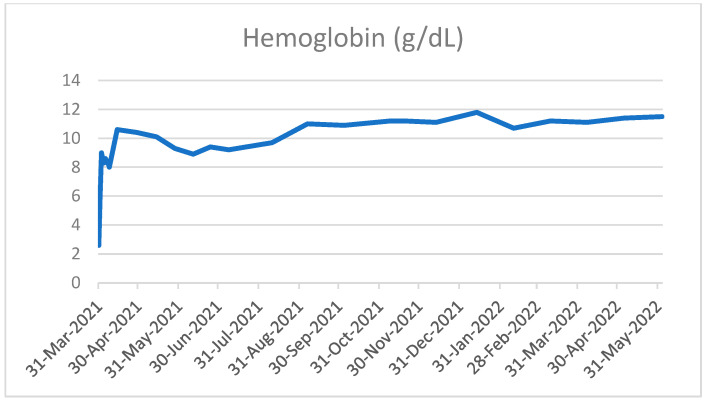
Trends of hemoglobin values over time.

Within two weeks of treatment start, the patient’s clinical condition markedly improved, hemoglobin levels rapidly recovered and stabilized (Figure 3) and she no longer needed oxygen therapy.

On August 2021, after four cycles of oncological treatment, the first re-evaluation with a TC scan showed partial response: in particular, the primitive tumor reduced up to 40 × 11 mm and pathological lymph-nodes also reduced in size. The ulceration completely disappeared and intact skin reconstituted (Figure 1b) without the necessity of locoregional treatments (e.g., radiotherapy or electrochemotherapy).

**Figure 4:**
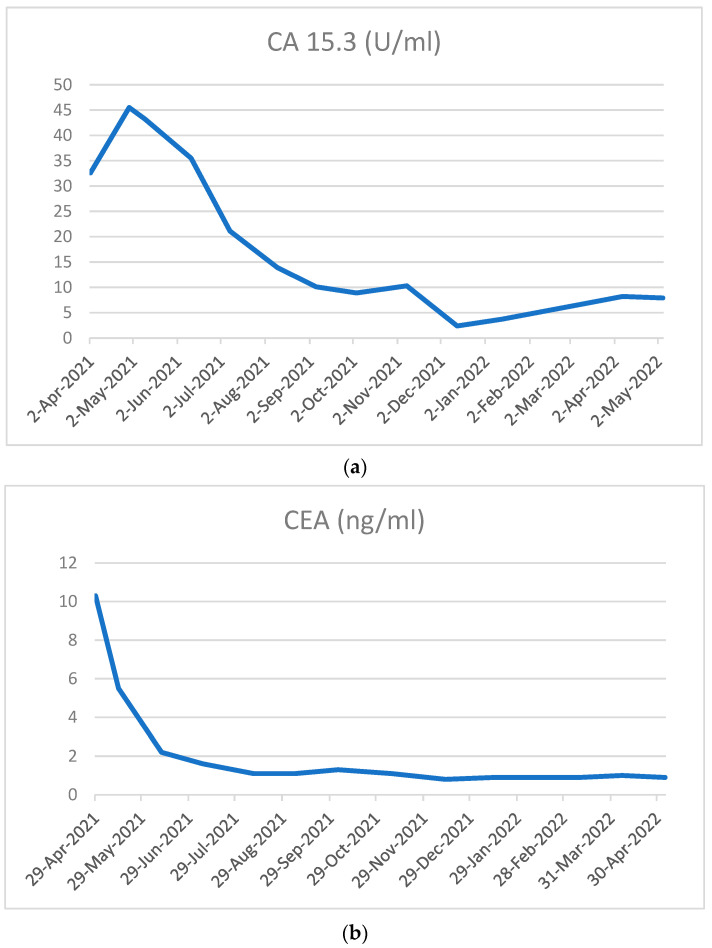
Trend of CA15.3 (**a**) and CEA (**b**) over time.

Subsequent re-evaluation with TC was performed in November 2021 after seven cycles of treatment and highlighted further response to treatment: the breast lesion had reduced to 36 × 8 mm, while the burden of bone disease remained stable in time (Figure 1c and Figure 2b). Concurrent decrease of neoplastic markers also proved the excellent response to treatment: the CEA reduced from to 10.9 ng/mL to 0.9 ng/mL, CA125 from to 443.7 U/mL to 12.7 U/mL and CA15.3 from to 32.6 U/mL to 3.7 U/mL at baseline and after seven cycles of treatment, respectively (Figure 2a,b and Figure 4). The oncological combination treatment was well tolerated over time and no major side effects were reported.

Currently, more than a year after diagnosis there is neither clinical nor radiological evidence of progression of disease and the patient is still receiving oncological treatment with clinical benefit and with excellent tolerance. 

## 3. Discussion

The advent of CDK4/6 inhibitors has revolutionized the treatment algorithm of advanced HR-positive and HER2-negative BC in both endocrine-sensitive and endocrine-resistant settings, becoming the recommended first-line treatment in association with ET, except for some life-threatening conditions like visceral crisis. 

The rapidity of response was crucial in our case presentation, given the continuous bleeding of the ulcerated breast lesion that caused severe and potentially life-threatening anemia, necessitating hospitalization for supportive therapies. Although the patient did not technically present a visceral crisis, this critical disease onset and the necessity of a rapid tumor shrinkage led us to consider the upfront use of CT. A paucity of direct comparisons between CDK4/6 inhibitors plus ET and CT as first-line treatment in luminal BC are available. 

A phase II study conducted in South Korea compared exemestane, ovarian function suppression plus Palbociclib with capecitabine in pre-menopausal women with progressive diseases after previous adjuvant or first-line tamoxifen, with 86% of the study population considered tamoxifen resistant. A significant longer median PFS was observed in the Palbociclib plus ET group (20.1 v.s. 14.4 months, HR = 0.66, *p* = 0.0235), with increased benefit in patients older than 35 years, with worse ECOG performance status, without visceral disease and not previously CT-treated. The ORR was similar in the two groups: 37% and 34% in Palbociclib plus ET and capecitabine arm, respectively. A post-hoc analysis showed no significant difference over time with respect to the best response in patients with measurable diseases, despite the fact that the median was slightly in favor of the capecitabine group (2.9 v.s. 4.3 months) [18].

The PEARL study is an interesting phase III trial comparing capecitabine and Palbociclib plus ET (with exemestane or fulvestrant) in post-menopausal patients with ER-positive and HER2-negative metastatic BC, resistant to aromatase inhibitors (AI). Unlike in the aforementioned Korean study, no statistical difference in PFS was observed between Palbociclib plus ET and capecitabine in this trial, neither in the full population nor in the Palbociclib plus fulvestrant group, which included the more appropriate endocrine companion in the AI-resistant setting. On the other hand, also in this study, a similar ORR was observed for Palbociclib plus fulvestrant (26.7%) and capecitabine (33.3%) treatment [19].

These results agree with the findings of the network meta-analysis of Giuliano et al., in which no CT regimen resulted in significantly superior PFS to Palbociclib plus letrozole in post-menopausal patients undergoing first- or second-line treatment. Also in terms of ORR, no CT schedule showed a higher proportion of patients achieving an overall response compared to Palbociclib and letrozole, except for weekly paclitaxel plus bevacizumab. However, paclitaxel and bevacizumab were not more active than other combinations of CDK4/6 inhibitors and ET, including Abemaciclib plus AI [20].

Another meta-analysis including PALOMA, MONALEESA and MONARCH studies confirmed a high grade of tumor regression with CDK4/6 inhibitors, without heterogeneity among the three different compounds: with an average of 55% in patients with AI sensitive and measurable diseases, this ORR demonstrated results superior to those observed with single agent CTs and quite comparable to those obtained with polyCT [21].

Although these studies did not systematically consider the rapidity of response, an exploratory analysis of the MONARCH-3 trial showed a 27.7% tumor size reduction after only two cycles of Abemaciclib and tumor shrinkage continued for at least 24 cycles, suggesting deeper and durable disease responses [8]. An early response was observed also with Ribociclib plus ET in the MONALEESA-2 trial, with a decreased tumor size in 78% of patients after 8 weeks of treatment [22]. Since the efficacy and response rates between ET plus CDK4/6 inhibitors and CT did not show a marked difference in the available literature data, evaluating the safety profile results is crucial in the treatment choice. In the Korean phase II study, a greater incidence of all grade hematological toxicities was observed in the CDK4/6 plus ET group against capecitabine, predominantly neutropenia (G3 75% v.s. 16%) without significant febrile neutropenia incidence. On the contrary, treatment-related serious adverse events (AEs) were more common in the CT group (17% versus 2%) [18].

Similarly, in the PEARL study, the incidence of G3 or more neutropenia was mostly observed in the Palbociclib plus ET group (57.4% with exemestane, 55.7% with fulvestrant and 5.5% in the capecitabine arm) with low febrile neutropenia incidence. On the other hand, non-hematologic G ≥ 3 toxicities were most frequent with capecitabine (38.8% versus 6.6% and 6.0% with Palbociclib plus exemestane and fulvestrant, respectively), as well as serious AEs and therapy discontinuation due to treatment-related toxicities. In addition, the median time to global health status deterioration was significantly longer in the CDK4/6 inhibitor group, with results of 8.6 months against 6.2 months in capecitabine treated patients (aHR = 0.67, *p* = 0.001), thus suggesting a better perceived quality of life [19].

Palbociclib, Ribociclib and Abemaciclib in association with ET have prolonged PFS versus ET alone in both endocrine and resistant settings in all pivotal clinical trials [5,6,7,8,9,10,11,23]. Piezzo et al., in their pooled and meta-analysis, confirmed a statistically significant improvement in PFS for each compound with a similar risk reduction amount and regarding the number of metastatic sites, the presence of visceral disease, the endocrine sensitivity and the TFI [21]. Similar results emerged from the Food and Drug Administration pooled analysis [24]. In addition, in the network meta-analysis of Giuliano et al., no significant difference in PFS and in the proportion of patients achieving an overall response was found among the three CDK4/6 inhibitors in association with AI or fulvestrant [20].

On the other hand, only Ribociclib and Abemaciclib have so far demonstrated a prolonged OS in association with ET. In the endocrine-sensitive setting, a significantly prolonged OS with Ribociclib associated with letrozole was observed in post-menopausal women in the MONALEESA 2 trial, with a 24% reduction in risk of death and a median survival of 63.9 months (v.s. 51.4 months; HR 0.76; *p* = 0.004) [25]. Similarly, in the endocrine-sensitive cohort treated with Ribociclib plus fulvestrant as first-line therapy in the MONALEESA 3 trial, the OS benefit was statistically confirmed in the last study update with a not-reached median and an HR of 0.64 [26]. A similar reduction in death risk was present also in pre- and peri-menopausal patients treated with Ribociclib plus ET (AI or tamoxifen) and goserelin in the MONALEESA 7 trial (HR 0.76 in overall population): of note, in patients with de novo disease and in patients aged less than 40 years the survival benefit observed with the CDK4/6 inhibitor over placebo was greater (HR 0.53 and 0.65, respectively) [27].

When considering endocrine-resistant patients, fulvestrant has demonstrated statistically increased survival when associated with Abemaciclib and Ribociclib in the MONARCH 2 and in the second-line treated cohort of the MONALEESA 3 trial, respectively. Although the enrolled population was different, since the MONARCH 2 trial included both pre- and post-menopausal patients, the amount of death risk reduction was similar (HR 0.757 in the MONARCH 2 trial and 0.780 in the endocrine-resistant cohort of the MONALEESA 3 study) [26,28].

These data are supported by the pooled and meta-analysis of Piezzo et al., including MONALEESA, MONARCH and PALOMA trials for which the OS data was available: a statistically significant reduction in risk of dying was observed in CDK4/6 inhibitor-treated patients with a pooled HR of 0.760 (*p* < 0.0001), regardless of the AI resistance or sensitivity. Furthermore, when grouped by type of CDK4/6 inhibitor, Ribociclib and Abemaciclib confirmed a statistically significant reduction in death risk while Palbociclib was the only class member not showing a statistical HR per OS. However, since the interaction test indicates that the differences may be ascribed to chance, these data should be interpreted with caution as differences in enrolled population, study design and subsequent or savage therapies might influenced the results [21].

Since these three CDK4/6 inhibitors have not been directly compared head-to-head in randomized clinical trials, the choice of the CDK4/6 inhibitor to associate with ET is influenced by differences in safety profile alongside the different schedule of administration and patient comorbidities. AEs are related to cell cycle arrest in highly proliferative tissues—e.g., hematopoietic and gastrointestinal—and thus these drugs are associated with anemia, leukopenia and neutropenia, especially with respect to CDK6 inhibition, that is particularly involved in hematopoiesis [29]. Of note, Abemaciclib has shown activity on other cyclin-dependent kinases and has higher selectivity for CDK4 over CDK6 and thus results in less hematological toxicity and increased gastrointestinal disorders, like nausea and diarrhea, which are also potentially mediated by CDK9 blockade [30]. Abemaciclib is also associated with reversible increased blood creatinine levels due to kidney tubular transporter inhibition without damaging glomerular function and therefore its use in renal impairment should be carefully evaluated [31]. Otherwise, QTc interval prolongation, hepatic toxicity and interstitial lung disease are concerning side effects mainly related to Ribociclib [31].

Therefore, in consideration of the non-substantial difference in terms of ORR and PFS between CDK4/6 inhibitor therapy and CT highlighted in the available literature data and considering the encouraging rapidity of responses observed, first-line treatment with a CDK4/6 inhibitor plus ET was chosen for the patient. The favorable safety profile of CDK4/6 inhibitors was another strength. Furthermore, the patient did not present with severe organ compromise as in proper visceral crisis, since bone marrow function was adequate and hemoglobin value stabilized after blood transfusions and appropriate local management of the breast ulcerated lesion.

## 4. Conclusions

Although no literature data about the efficacy and the safety of CDK4/6 inhibitors in visceral crises are available, these drugs have demonstrated high response rates and notable disease control in several studies. In the few examples of direct comparison data, CDK4/6 inhibitors plus ET have shown comparable ORR to those obtained with mono-CT, with a more favorable toxicity profile. Of note, in the MONARCH 3 trial evaluating Abemaciclib plus AI in post-menopausal woman with endocrine-sensitive disease, the ORR in patients with measurable diseases reached 61%, with a median DOR of 32.7 months [32]. Therefore, our case report also endorses the upfront use of CDK4/6 inhibitor combinations in critical clinical situations in the absence of severe organ dysfunction or rapid disease progression and after multidisciplinary discussion.

## Data Availability

Not applicable.
